# High risk of multiple gastric cancers in Japanese individuals with Lynch syndrome

**DOI:** 10.1002/ags3.12809

**Published:** 2024-04-22

**Authors:** Nobuhiko Kanaya, Thijs A. van Schaik, Hideki Aoki, Yumiko Sato, Fumitaka Taniguchi, Kunitoshi Shigeyasu, Kokichi Sugano, Kiwamu Akagi, Hideyuki Ishida, Kohji Tanakaya

**Affiliations:** ^1^ Department of Surgery National Hospital Organization Iwakuni Clinical Center Yamaguchi Japan; ^2^ Department of Neurosurgery Brigham and Women's Hospital, Harvard Medical School Boston Massachusetts USA; ^3^ Department of Gastroenterological Surgery Okayama University Graduate School of Medicine, Dentistry and Pharmaceutical Sciences Okayama Japan; ^4^ Division of Tumor Biology and Immunology The Netherlands Cancer Institute, Oncode Institute Amsterdam The Netherlands; ^5^ Department of Pathology National Hospital Organization Iwakuni Clinical Center Yamaguchi Japan; ^6^ Department of Genetic Medicine Kyoundo Hospital, SSasaki Foundation Tokyo Japan; ^7^ Division of Molecular Diagnosis and Cancer Prevention Saitama Cancer Center Saitama Japan; ^8^ Department of Digestive Tract and General Surgery, Saitama Medical Center Saitama Medical University Kawagoe Japan

**Keywords:** cumulative risk, gastric cancer, Japanese individuals, Lynch syndrome, multiple gastric cancers

## Abstract

**Aim:**

Lynch syndrome (LS) is a dominantly inherited syndrome characterized by an increased risk for LS associated tumors such as colorectal cancer (CRC) and gastric cancer (GC). However, the clinical benefit of surveillance for GC remains unclear while it has already been recommended for CRC. This study aimed to elucidate the clinical features of GC in Japanese individuals with LS, and the risk of developing multiple GCs to build regional‐tailored surveillance programs in LS patients with GC.

**Methods:**

Data on Japanese individuals with LS were retrospectively collected from a single institution. The clinical features of GC, including the cumulative risk of multiple GCs, were analyzed.

**Results:**

Among 96 individuals with LS (*MLH1*/*MSH2*/*MSH6*, 75:20:1), 32 GC lesions were detected in 15 individuals with LS (male/female, 11:4). The median age at initial GC diagnosis was 52.7 y (range: 28–71). Histological examination revealed a predominance of intestinal type (19/24: 87.5%). Moreover, the majority of the GC lesions (82%) were determined to have high‐frequency of microsatellite instability. The cumulative risk of individuals with LS developing GC at 70 y was 31.3% (*MLH1* 36.1%, *MSH2* 18.0%). Notably, the cumulative risk of individuals with LS developing metachronous and/or synchronous GCs at 0, 10 and 20 y after initial diagnosis of GC was 26.7%, 40.7%, and 59.4%, respectively.

**Conclusion:**

Due to a higher risk of developing multiple GCs, intensive surveillance might be especially recommended for Japanese individuals with LS associated initial GC.

## Graphical abstract

Multiple gastric cancers can be a risk factor in Lynch syndrome. Intensive surveillance might be recommended for Japanese individuals with LS associated with *MLH1* or *MSH2* variants.

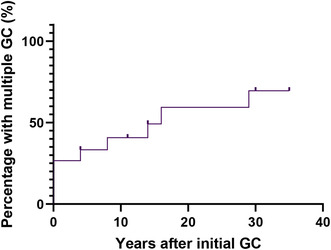

## 1 INTRODUCTION

Gastric cancer (GC) is the fourth most common cancer in the world and remains the second most frequent cause of cancer‐related deaths.^1^ These incidences differ greatly between geographical environment factors. For example, the observed incidences in Eastern Europe, South America, and East Asia including Japan are far higher than in North America.^2^ In Japan, the estimated lifetime cumulative risk of sporadic GC is 11.4% and 5.7% in males and females, respectively.^3^ The main risk factors for developing GC are *Helicobacter pylori* infection, smoking, alcohol consumption, obesity, and gastroesophageal reflux.^4^ In addition, several genetic factors such as hereditary diffuse GC, Li‐Fraumeni syndrome, and Lynch syndrome (LS) have been reported to increase the risk of GC.^5^

LS is a dominantly inherited syndrome generally caused by a pathogenic germline variant in one of the mismatch repair (MMR) genes^6^, ^7^ and epithelial cell adhesion molecule (*EPCAM*). Approximately 1 in 250–1000 of the general population carry a mutation in an MMR gene.^7^, ^8^ Deficiency in the MMR genes can cause microsatellite instability (MSI), resulting in molecular abnormalities that are frequently observed in tumors associated with LS.^9^ Colorectal cancer (CRC) and endometrial cancer (EC) are the most commonly diagnosed tumors in individuals with LS.^10^ Despite previous LS studies showing that the cumulative risk of developing GC is lower compared to CRC, this risk greatly varies between geographical regions.^11^, ^12^ For example, the cumulative risk for sporadic GC development in some Asian countries, such as Japan and South Korea, is considerably higher than in Western European countries such as the Netherlands.^13^

In Japan, the incidence of synchronous and metachronous sporadic GCs are 6%–14% and 2.7%–15.6%, respectively.^14^ To ensure early detection of multiple GCs in patients with sporadic GC, it is recommended to undergo endoscopic surveillance after the initial diagnosis.^15^

However, little is known about the development of multiple GCs in individuals with LS, and particularly the differences between geographical regions with dissimilar GC incidence rates. The current guidelines for surveillance and surgical option of GC in individuals with LS were mainly built with data from Western countries and can thus be insufficient for other parts of the world. Therefore, elucidation of the clinical features will provide meaningful insights to develop regional‐tailored surveillance programs in LS patients. This study aims to elucidate the clinical features of GC in Japanese individuals with LS, and the risk of developing multiple GCs.

## 2 MATERIALS AND METHODS

### 2.1 Families, samples, and data collection

A retrospective single‐institution study of LS individuals in Japan was conducted from January 2003 to December 2021 at Iwakuni Clinical Center. Individuals underwent genetic testing if they met the revised Bethesda guidelines,^16^ including cancer with a high level of MSI, or a modified version of the Amsterdam II Criteria,^17^ which included GC as one of the LS‐associated tumors, as it is common in Asian individuals with LS.

In this study we analyzed individuals with MMR gene variants and the obligation carriers. Clinical information included sex, age at the diagnosis of GC, location of the GC, histological type, and stage (Union for International Cancer Control (UICC)‐8). The clinical information was obtained from either medical records or directly from the individuals who received genetic counseling. Metachronous or synchronous GC was defined as a secondary GC diagnosed more than 12 mo after the initial diagnosis of primary GC. In our hospital, individuals desiring or considering genetic testing are generally provided genetic counseling by full‐time clinical geneticists with Board‐certified clinical geneticists at the Japanese Board of Medical Genetics and Genomics, Clinical Genetics, or part‐time certified genetic counselors. Therefore, the medical system had already been established for individuals who provided written informed consent for genetic testing.

### 2.2 Microsatellite instability analysis

MSI analysis was performed on paraffin‐embedded tumor specimens resected by surgery or endoscopy. To detect loss of function in MMR, MSI was evaluated by FALCO Biosystems (Kyoto, Japan) for five mononucleotide repeat markers (NR21, NR24, BAT25, BAT26, and MONO27), or (BAT 25, BAT26, D2S123, D5S346, and D17S250). Tumors showing an allelic shift of two or more loci were defined as high‐frequency of microsatellite instability (MSI‐H), those with an allelic shift of one locus as low MSI (MSI‐L), and those with no allelic shift at any marker as microsatellite stable (MSS).

### 2.3 Germline mutation analyses

Genomic DNA was extracted from peripheral blood samples using the standard phenol extraction/purification procedure. Germline mutation analyses were performed by sequencing the entire coding region in the *MLH1*, *MSH2*, and *MSH6* genes. Polymerase chain reaction (PCR) products were purified with ExoSAP‐IT (Affymetrix, Santa Clara, CA, USA) and underwent capillary sequencing using a BigDye Terminator v1.1 Cycle Sequencing Kit (Applied Biosystems, Foster City, CA, USA). Subsequently, these products were purified using a BigDye XTerminator Purification Kit (Applied Biosystems) and loaded into a 3730xl Genetic Analyzer (Applied Biosystems). The detection of genomic rearrangements such as deletion or duplication was done by multiplex ligation‐dependent probe amplification (MLPA) performed using a SALSA MLPA MLH1/MSH2 Kit (MRC‐Holland, Amsterdam, The Netherlands) in accordance with the manufacturer's protocol.^18^ From 2015 onwards, mutation analysis of all the coding exons and intron‐exon boundaries of 19 known genes (*bone morphogenetic protein receptor type 1A, cadherin 1, epithelial cell adhesion molecule, MBD4, MLH1, mutL homolog 3, MSH2, MSH3, MSH6, MUTYH, PMS1 homolog 1, mismatch repair system component, PMS2, POLD1, POLE, phosphatase and tensin homolog, SMAD4, serine/threonine kinase 11, transforming growth factor‐β receptor 2 and tumor protein p53* [*TP53]*) and the entire genomic sequence of *adenomatous polyposis coli (APC)* was performed by massive parallel sequencing analysis with a MiSeq sequencer (Illumina, San Diego, CA, USA). WES analysis was conducted by Novogene (China) via Chemical Dojin (Kumamoto, Japan), as follows: The Agilent SureSelect Human All Exon V6 (58 m) kit (Agilent Technologies, Santa Clara, CA, USA) was used for the DNA target enrichment, followed by sequencing with an Illumina HiSeq4000 sequencer (Illumina). When a pathogenic mutation of the MMR gene was detected, the patient was diagnosed with LS.

### 2.4 Statistical analysis

The cumulative risk of developing GC, and multiple GCs, was analyzed by a Kaplan–Meier plot. Data are presented as totals, median (range), or percentages. All statistical analyses were performed using JMP software (v. 14, SAS Institute, Cary, NC, USA). The log‐rank test was used for Kaplan–Meier survival analyses. *P* values <0.05 were considered significant.

## 3 RESULTS

### 3.1 Identified variant in the *MLH1* and *MSH2* genes

**FIGURE 1:**
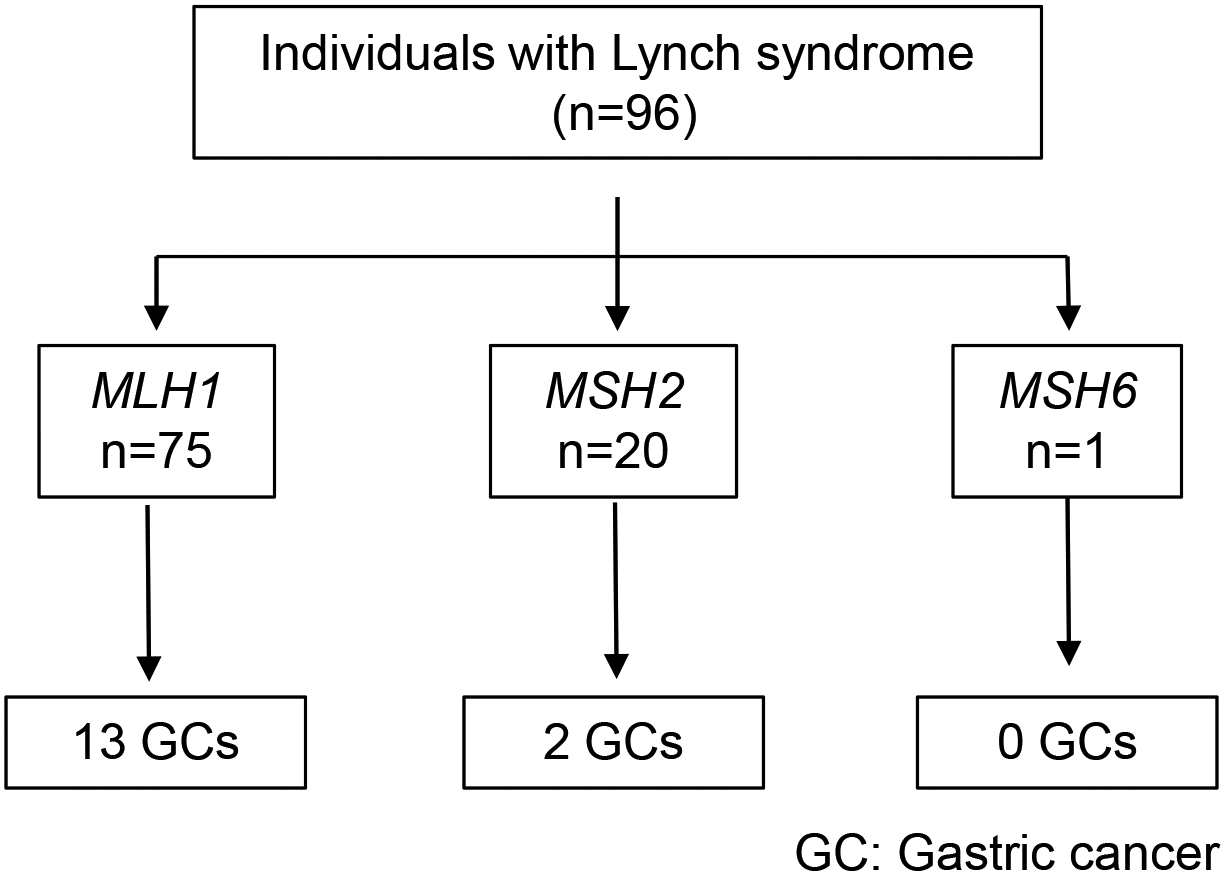
Flow diagram for gastric cancer in Lynch syndrome. GC, gastric cancer.

Thirty‐one probands were diagnosed with LS (Table S1). In the 31 probands, 18 probands had an *MLH1* gene variant, 12 probands had an *MSH2* gene variant, and one proband had an *MSH6* gene variant. In total, 96 individuals (*MLH1*: 75, and *MSH2*: 20, and *MSH6*: 1) were diagnosed with LS in their families (Table 1; Figure 1).

**TABLE 1:** Background of individuals with Lynch syndrome.

Individuals	*MLH1*	*MSH2*	*MSH6*	Total
Total	75	20	1	96
Male	36	8	1	45
Female	39	12	0	51

Abbreviation: MMR, mismatch repair.

### 3.2 Clinical features of the GC in the LS individuals

After testing for LS in the families of the 31 probands, 15 individuals with GC and an MMR variant were found (Tables 2 and 3), with a male‐to‐female ratio of 11:4 and the ratio of MMR gene variants (*MLH1*:*MSH2*) was 13:2. Furthermore, the median age at diagnosis of the initial GC was 52.7 (range: 28–71) y (male/female, 54 [range: 28–71]: 61 [range: 49–67]). The GC lesions were diagnosed in the lower stomach (28%, 9/32), 25% in the upper stomach (8/32), 25% in the middle stomach (8/32), and 22% the data were unknown (7/32). *H. pylori* infection status was not confirmed in this study. The available histologic classification (Lauren's criteria) of GC showed that intestinal type accounted for 87.5% (21/24) of the cancers, while diffuse type accounted for the remaining 12.5% (3/24).

**TABLE 2:** Clinicopathological characteristics of individuals with gastric cancer in Lynch syndrome.

	*MLH1*	*MSH2*	Total
Individuals	13	2	15
Gender			
Male	9	2	11
Female	4	0	4
Age at initial GC (y)	54.5	41.5	52.7 (28–71)
Location of initial GC (lesions)			
Upper	7	1	8
Middle	8	0	8
Lower	8	1	9
Unknown	5	2	7
Multiple GC (individuals)			
Synchronous	6	0	6
Metachronous	5	2	7
Histological type			
Intestinal	19	2	21 (87.5%)
Diffuse	3	0	3 (12.5%)
Unknown	6	2	8
UICC Stage			
I	17	1	18
II	3	0	3
III	2	1	3
IV	0	0	0
Unknown	6	2	8
Multiple primary cancers (individuals)			
Colon	9	1	10 (67%)
Stomach	4	1	5 (33%)
MSI			
MSI‐H	7	2	9
MSS, MSI‐L	2	0	2
Unknown	21	2	23

Abbreviations: GC, gastric cancer; MSI, microsatellite instability; MSI‐H, MSI‐high; tub, tubular adenocarcinoma; poor, poorly differentiated adenocarcinoma; sig, signet‐ring cell carcinoma.

**TABLE 3:** Case reports with gastric cancer‐associated Lynch syndrome.

Case	Gender	MMR gene	Initial cancer	Age (y) at initial cancer	Age	Location	Macroscopic type	histological type	Stage	MSI
1	Female	*MLH1*	CRC	34	60	n.d.	2	n.d.	n.d.	n.d.
2	Female	*MLH1*	CRC	41	49	L	2	Intestinal	II	n.d.
					78	U	2	Intestinal	IIIA	MSI‐H
3	Male	*MLH1*	CRC	50	67	U	2	Intestinal	IIB	N/A
					67	M	0‐llc	Intestinal	IA	n.d.
4	Male	*MLH1*	GC	30	30	n.d.	n.d.	n.d.	n.d.	n.d.
5	Male	*MLH1*	CRC	44	44	M	0‐llc	Intestinal	IA	n.d.
					58	U	0‐lla	Diffuse	IA	n.d.
6	Male	*MLH1*	GC	35	35	n.d.	n.d.	n.d.	n.d.	n.d.
7	Male	*MLH1*	CRC	37	54	L	0‐I	Intestinal	IA	n.d.
					59	M	0‐llc + IIa	Diffuse	IB	N/A.
8	Male	*MLH1*	CRC	34	58	L	3	Intestinal	IIIA	MSI‐H
					58	M	2	Intestinal	IIA	n.d.
9	Male	*MLH1*	GC	54	54	U	1	Intestinal	IB	n.d.
					62	M	0‐I	Intestinal	IA	MSI‐H
					62	L	0‐llb	Intestinal	IA	MSI‐H
					62	L	0‐llb	Intestinal	IA	n.d.
10	Male	*MSH2*	GC	28	28	n.d.	n.d.	n.d.	n.d.	n.d.
					44	n.d.	n.d.	n.d.	n.d.	n.d.
11	Male	*MLH1*	GC	57	57	n.d.	n.d.	n.d.	n.d.	n.d.
					57	n.d.	n.d.	n.d.	n.d.	n.d.
12	Male	*MLH1*	CRC	65	71	M	0‐llc	Diffuse	IA	MSS
					71	M	0‐lla	Intestinal	IA	MSS
13	Female	*MLH1*	CRC	45	67	L	0‐lla + llc	Intestinal	IA	MSI‐H
					67	M	0‐llb	Intestinal	IA	n.d.
					67	L	0‐llb	Intestinal	IA	n.d.
					67	L	0‐llb	Intestinal	IA	n.d.
					70	U	0‐lla	Intestinal	IA	MSI‐H
					77	U	0‐I	Intestinal	IA	MSI‐H
14	Female	*MLH1*	CRC	36	62	U	n.d.	n.d.	n.d.	n.d.
15	Male	*MSH2*	CRC	46	55	L	2	Intestinal	IIIC	MSI‐H
					59	U	0‐I	Intestinal	IA	MSI‐H

Abbreviations: L, lower; M, middle; MSI‐H, MSI‐high; MSI‐L, MSI‐low; MSS, microsatellite stable; muc, mucinous adenocarcinoma. n.d., no data; N/A, not available; poor, poorly differentiated adenocarcinoma; tub, tubular adenocarcinoma; U, upper; MSI, microsatellite instability.

The distribution of the UICC stages of the 24 GC lesions was: stage I, 75% (18/24); stage II, 12.5% (3/24); stage III, 12.5% (3/24); and stage IV, 0% (0/24). Interestingly, out of individuals with GC, the incidences of synchronous and/or metachronous GCs were 40% (6/15) and 46.7% (7/15), respectively, and MSI‐H accounted for 82% (9/11) of the available specimens.

### 3.3 Surveillance and outcome for cases with LS‐associated GC

**FIGURE 2:**
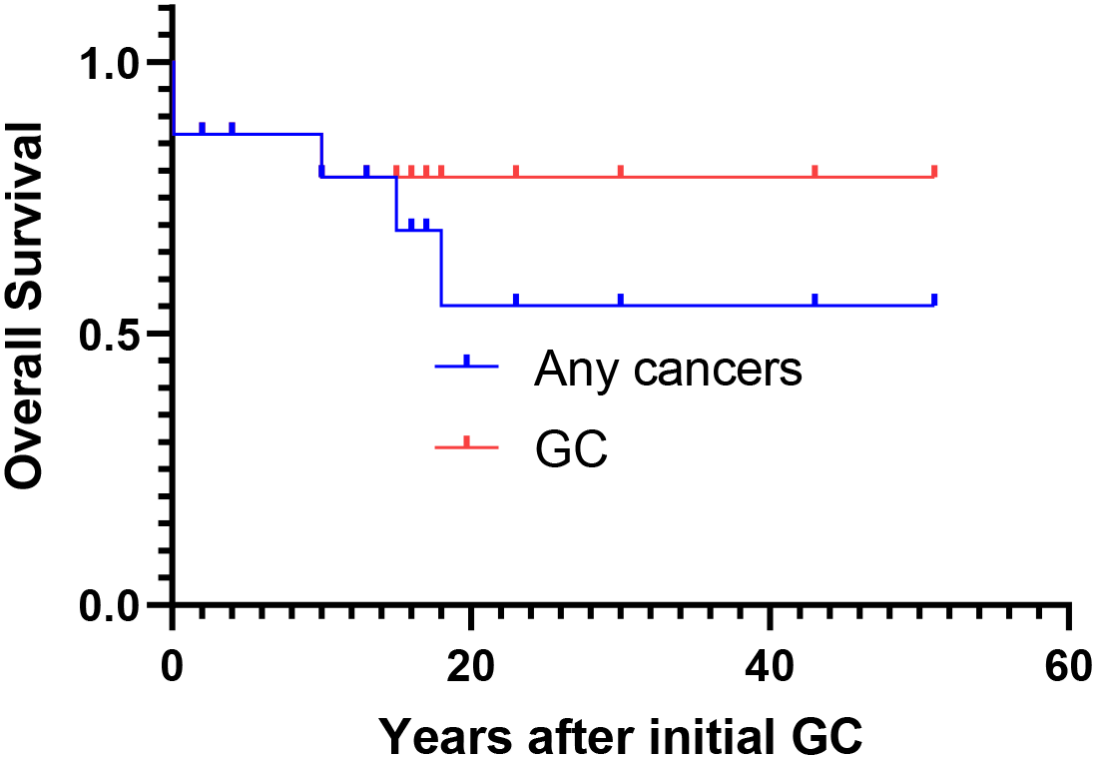
Cancer‐associated overall survival of patients with gastric cancer. GC, gastric cancer.

Evidence supporting the beneficial effects of surveillance for LS‐associated GC remains limited; however, esophagogastroduodenoscopy (EGD) is proposed by the guidelines.^19^, ^20^ In our institution, EGD has been performed with an interval of every 6–20 mo (Table 4). Out of the 17 GC lesions with available surveillance data, nine GC lesions were detected by surveillance, while eight GC lesions were detected differently. Moreover, the majority of GC detected with surveillance (89%, 8/9 GC lesions) was in an early stage (IA or IB), while the majority of GC detected without surveillance (50%, 4/8 lesions) were at stage II‐IIIC. Furthermore, the only patient with a 20‐mo interval EGD had stage IIIA. Among 15 individuals with GC, 5‐y GC related overall survival was 86.7% (Figure 2).

**TABLE 4:** Surveillance, treatment and outcome for cases with gastric cancer‐associated Lynch syndrome.

Case	Gender	Age	Stage	Surveillance	Duration from previous EGD (mo)	Treatment	Outcome	Endpoint (y)
1	Female	60	n.d.	No		Distal gastrectomy	Dead (trauma)	90
2	Female	49	II	No		Distal gastrectomy	Dead (senility)	90
		78	IIIA	Yes	20	Total gastrectomy		
3	Male	67	IIB	No		Proximal gastrectomy	Dead (CRC)	85
		67	IA					
4	Male	28	n.d.	n.d.	n.d.	n.d.	Dead (GC)	28
5	Male	44	IA				Dead (CRC)	59
		58	IA	Yes		Endoscopic resection		
6	Male	35	n.d.	n.d.	n.d.	n.d.	Dead (GC)	35
7	Male	54	IA	No		Proximal gastrectomy	Alive	71
		59	IB	Yes	12	Total gastrectomy		
8	Male	58	IIIA	No		Distal gastrectomy	Alive	74
		58	IIA					
9	Male	54	IB	No		Proximal gastrectomy	Alive	77
		62	IA	Yes	12	Distal (total) gastrectomy		
		62	IA					
		62	IA					
10	Male	28	n.d.	n.d.	n.d.	n.d.	Dead (pneumonia)	71
		44	n.d.	n.d.	n.d.	n.d.		
11	Male	57	n.d.	n.d.	n.d.	n.d.	Dead (GC)	67
		57	n.d.		n.d.	n.d.		
12	Male	71	IA	Yes	4–14	n.d.	Alive	73
		71	IA	Yes				
13	Female	67	IA	No		Distal gastrectomy	Alive	
		67	IA					
		67	IA					
		67	IA					
		70	IA	Yes	1–19	Endoscopic resection		
		77	IA	Yes	6–21	Endoscopic resection		77
14	Female	62	n.d.	n.d.	n.d.	n.d.	Alive	75
15	Male	55	IIIC	No		Distal gastrectomy	Alive	
		59	IA	Yes	12	Endoscopic resection		60

Abbreviations: EGD, esophagogastroduodenoscopy; n.d., no data.

### 3.4 Cumulative risk of developing GC

**FIGURE 3:**
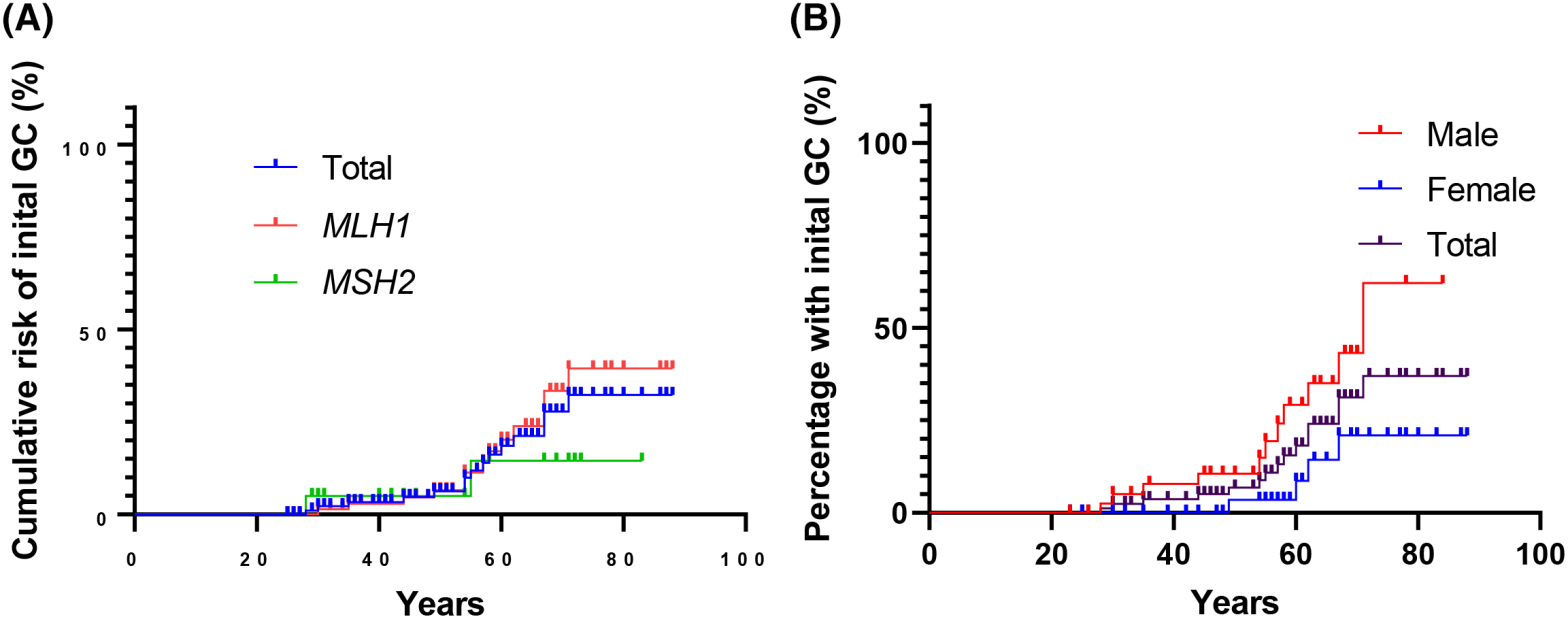
The cumulative risk of developing gastric cancer. (A) Difference between MMR gene mutation. (B) Difference between gender. GC, gastric cancer; MMR, mismatch repair.

**FIGURE 4:**
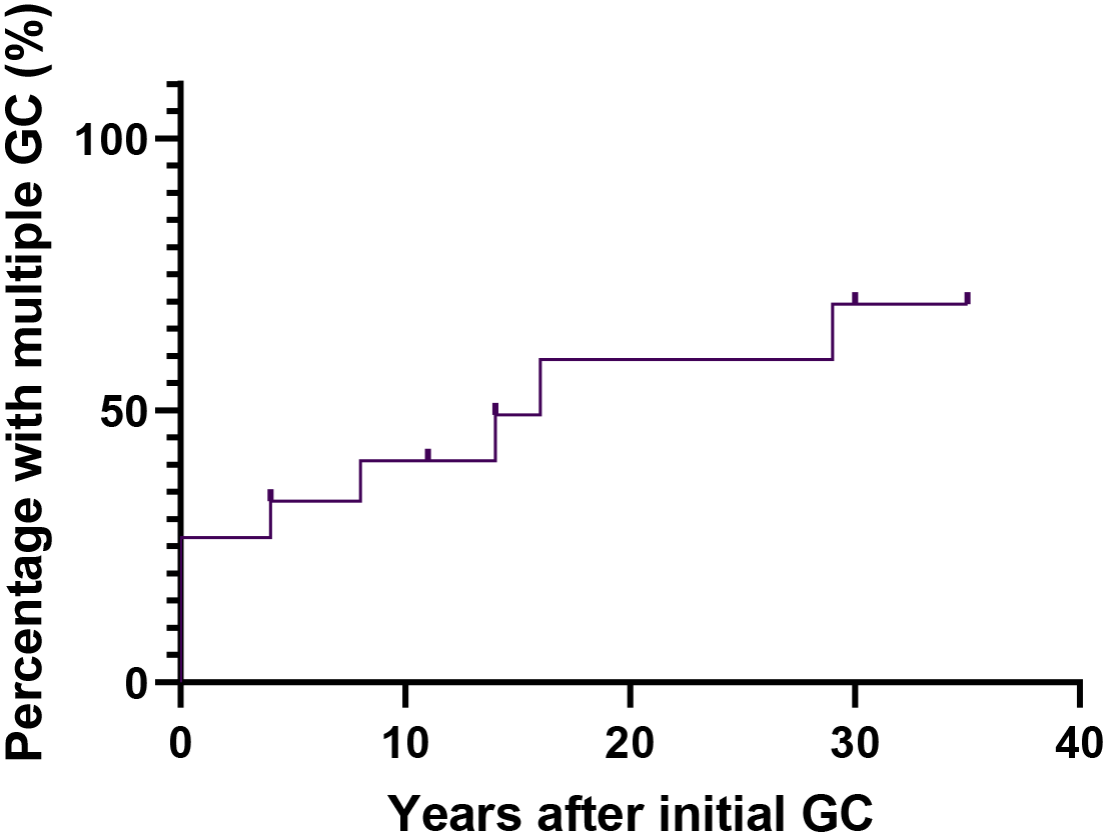
The cumulative risk of developing synchronous and/or metachronous gastric cancer. GC, gastric cancer.

The cumulative risk of individuals developing GC at 60, 70, and 75 y was 18.2% (*MLH1* 18.9%, *MSH2* 18.0%), 31.3% (*MLH1* 36.1%, *MSH2* 18.0%), and 37% (*MLH1*: 42.5%, *MSH2*: 18%), respectively (Figure 3A). This cumulative risk for males was significantly higher than for females (*P* = 0.0472) (Figure 3B). Interestingly, the cumulative risk of developing synchronous and/or metachronous GCs at 0 y, 10 y, and 20 y was 26.7%, 40.7%, and 59.4%, respectively (Figure 4).

## 4 DISCUSSION

In this study we explored the clinical and molecular features of GC in a cohort of Japanese individuals with LS. Within this cohort, 15 MMR gene‐variant individuals were diagnosed with a total of 32 GC lesions, and this tumor development was predominantly observed in males. Moreover, the age of GC diagnosis in individuals with LS was lower compared to sporadic GC in Japan.^21^ In our cohort, the majority of GC associated with LS had MSI‐H and there was a higher risk for synchronous and/or metachronous GCs compared to the sporadic GCs.^22^ These results will help physicians to develop appropriate surveillance strategies tailored to their patient cohort for early detection of synchronous and/or metachronous GC lesions.

In Japan and other regions of the world, GC in LS is characterized by male dominance, early age at onset, and high *MLH1* or *MSH2* variant incidence..^13^, ^23^ Moreover, in this subset of GCs the intestinal type is predominantly the pathological type compared to the diffuse type.^24^, ^25^ Together, these studies show that our results are consistent with previously published data in East Asian countries.

Furthermore, MSI analysis of the available GC lesions revealed that 82% of the lesions are MSI‐H. These findings are in accordance with previously published studies.^26^ In sporadic GC, MSI‐H incidence was 5.6%–8% in Asian countries, while 7%–24% in Western countries.^27^ Since MSI‐H is found in 90% of CRC and 41.7%–100% of EC in LS‐associated tumors, it is suggested to be a hallmark for LS‐associated tumors.^28^ Therefore, many guidelines recommend universal MSI screening for individuals with CRC or EC. Thus, the revised Bethesda criteria has been used for primary screening to detect LS in individuals with CRC. In our study, all individuals with GC met these further revised Bethesda criteria, where CRC is replaced with GC. The further revised version can be useful for individuals with GC to examine the likelihood of LS, after which the individual can undergo MSI analysis after genetic counseling.

The cumulative risk of GC in LS cases at 70 y of age in Japan (14.5%–41%) is much higher than in Western countries (6%–13%).^13^, ^24^, ^25^ This higher risk of individuals with LS in Japan displays the importance of regionally managing GC.^29^ In Western countries, individuals with GC in LS show an average 5‐y survival rate of 61%, which could potentially be increased with appropriate surveillance and treatment plans.^30^ In our study the cumulative risk of individuals developing GC at 70 y was 31.3% (*MLH1* 36.1%, *MSH2* 18.0%). These data are higher compared to previous reports in both Western and Asian reports, which might be caused by the detection of GC by surveillance for individuals with LS.^13^, ^24^, ^25^ Additionally, the cumulative risk for individuals with the *MLH1* variant was higher than with the *MSH2* variant, while a previous study in Korea showed both variants are higher risk factors to develop GC for individuals with LS.^23^ The result might be caused by the dominance of individuals with the *MLH1* variant. Thus, further investigations could be interesting to confirm the fact.

Moreover, a higher risk of developing multiple tumors, including metachronous and spontaneous cancers, is found in individuals with LS.^19^, ^20^ Previous studies have revealed the high risk for multiple tumors in LS patients with CRC. Consequently, colonoscopy surveillance was introduced, which reduced the overall mortality of CRC in LS patients by ~65%.^20^, ^31^ The cumulative risk in synchronous and/or metachronous GCs with LS remains unknown in both Japan and the rest of the world; and therefore, adequate follow‐up screening and treatment protocols are absent. In our study, the cumulative risk of LS patients developing synchronous and/or metachronous GCs at 0, 10, and 20 y after diagnosis of the initial GC were 26.7%, 40.7%, and 59.4%, respectively. Our results suggest that the development of synchronous and/or metachronous GCs in individuals with LS is more frequent than in patients with sporadic synchronous and/or metachronous GCs.^32^ The cumulative risk is comparable to metachronous CRCs in Western countries, and thus requires similar management. In Japan, screening such as radiography or EGD have originally been performed to detect sporadic GC from the age of 50 y.^33^ However, several guidelines, such as the European Society of Digestive Oncology, US Multi‐Society Task Force, and NCCN suggested that EGD (every 2–4 y) is recommended in individuals with LS from the age of 30–40 y.^27^, ^29^, ^34^ In this study, 60% (9/15) of the patients who underwent initial GC surgery voluntarily enrolled in our surveillance program. This surveillance resulted in the detection of 71% (5/7) of the metachronous GCs, the majority of which were detected at an early stage by EGD. Together, these observations suggest that it is critical to consider multiple GCs after detection of initial GC in individuals with LS and that surveillance might have tremendous clinical benefits in individuals with LS by the early detection of GC. Considering this study, we propose individuals with LS should have surveillance by EGD (every 1–3 y) from the age of 30–35 y and testing of HP injection, and then, individuals with LS‐associated initial GC should have surveillance with EGD (every year) after the initial GC.

Additionally, in some LS‐associated cancers including CRCs and ovarian cancer (OC) risk‐reducing surgery is performed; it includes extended resection to reduce the risk of developing initial CRC or OC and metachronous tumors.^27^ However, no data are available to support this treatment in GC. The risk‐reducing surgery, such as extended total gastrectomy, can reduce the risk of developing metachronous tumors, but can lead to other complications such as body weight loss, reflux esophagitis, and anemia.^35^ Due to no evidence of risk‐reducing surgery GC, it is critical to build evidence of surgical options including extended total gastrectomy as well as standard gastrectomy according to the situation of an individual with GC such as their ages, life philosophy, and quality of life after surgery.

This study has several limitations, including the small initial sample size (individuals with LS‐associated GC, *n* = 15), the majority of *MLH1 and MSH2* variant, and its retrospective single‐center design. Then the clinical features of LS‐associated GC should be investigated by prospective and multiple‐center design and, further, it is critical to build evidence for useful surveillance and risk‐reducing surgery to improve overall survival of LS‐associated GC.

## 5 CONCLUSION

The present study aimed to determine the clinical features and cumulative risk of synchronous and/or metachronous GCs neoplasms of Japanese patients with LS‐associated GC. Although limited data from Eastern Asia are available, our results suggest that multiple GCs can be a risk factor in LS and, therefore, intensive surveillance might be recommended for Japanese individuals with LS‐associated with *MLH1* or *MSH2* variants.

## AUTHOR CONTRIBUTIONS

K.T. and N.K. designed the study. K.T., N.K., F.T., and K. Shigeyasu collected the data. N.K., K.T., and T.V.S. wrote the draft and analyzed the data. H.A. performed treatment for the individuals. M.T. performed pathological diagnosis. K. Sugano, K.A., and H.I. conducted genetic testing. K.T. is the guarantor of this work and, as such, had full access to all the data in the study and takes responsibility for the integrity of the data and the accuracy of the data analysis.

## FUNDING INFORMATION

This research was supported in part by the Dial Study from the Japan Agency for Medical Research and Development, AMED. The research was also supported by Japan AMED under the grant reference; JP 18kk0205004 and JSPS KAKENHI grant reference; JP18K07339.

## CONFLICT OF INTEREST STATEMENT

K.A. had a lecture fee from Merck Sharp & Dohme (MSD). The other authors declare no conflicts of interest for this article.

## ETHICAL APPROVAL

Approval of the research protocol: The study was approved by the institutional review board of the Iwakuni Clinical Center (No. 2774).

Informed Consent: Before the genetic testing, the individual received genetic counseling from clinical geneticists and gave written informed consent.

Registry and the Registration No. of the study/Trial: N/A.

Animal Studies: N/A.

## Supporting information


Table S1.

## References

[1] Japanese Gastric Cancer Association . Japanese gastric cancer treatment guidelines 2014 (ver. 4). Gastric Cancer. 2017;20(1):1–19. 10.1007/s10120-016-0622-4 PMC5215069 27342689

[2] Machlowska J , Baj J , Sitarz M , Maciejewski R , Sitarz R . Gastric cancer: epidemiology, risk factors, classification, genomic characteristics and treatment strategies. Int J Mol Sci. 2020;21(11):4012. 10.3390/ijms21114012 PMC7312039 32512697

[3] Ferlay J , Shin HR , Bray F , Forman D , Mathers C , Parkin DM . Estimates of worldwide burden of cancer in 2008: GLOBOCAN 2008. Int J Cancer. 2010;127(12):2893–2917. 10.1002/ijc.25516 21351269

[4] Cavaleiro‐Pinto M , Peleteiro B , Lunet N , Barros H . *Helicobacter pylori* infection and gastric cardia cancer: systematic review and meta‐analysis. Cancer Causes Control. 2011;22(3):375–387. 10.1007/s10552-010-9707-2 21184266

[5] Ishida H , Yamaguchi T , Tanakaya K , Akagi K , Inoue Y , Kumamoto K , et al. Japanese Society for Cancer of the colon and Rectum (JSCCR) guidelines 2016 for the clinical practice of hereditary colorectal cancer (translated version). J Anus Rectum Colon. 2018;2(Suppl I):S1–s51. 10.23922/jarc.2017-028 31773066 PMC6849642

[6] Lynch HT , de la Chapelle A . Hereditary colorectal cancer. N Engl J Med. 2003;348(10):919–932. 10.1056/NEJMra012242 12621137

[7] Boland CR , Shike M . Report from the Jerusalem workshop on Lynch syndrome‐hereditary nonpolyposis colorectal cancer. Gastroenterology. 2010;138:2197‐e1. 10.1053/j.gastro.2010.04.024 PMC3032350 20416305

[8] Tanakaya K . Current clinical topics of Lynch syndrome. Int J Clin Oncol. 2019;24(9):1013–1019. 10.1007/s10147-018-1282-7 29744602

[9] Latham A , Srinivasan P , Kemel Y , Shia J , Bandlamudi C , Mandelker D , et al. Microsatellite instability is associated with the presence of Lynch syndrome pan‐cancer. J Clin Oncol. 2019;37(4):286–295. 10.1200/jco.18.00283 30376427 PMC6553803

[10] Karimi P , Islami F , Anandasabapathy S , Freedman ND , Kamangar F . Gastric cancer: descriptive epidemiology, risk factors, screening, and prevention. Cancer Epidemiol Biomarkers Prev. 2014;23(5):700–713. 10.1158/1055-9965.epi-13-1057 24618998 PMC4019373

[11] Karimi M , von Salomé J , Aravidis C , Silander G , Askmalm MS , Henriksson I , et al. A retrospective study of extracolonic, non‐endometrial cancer in Swedish Lynch syndrome families. Hered Cancer Clin Pract. 2018;16:16. 10.1186/s13053-018-0098-9 30386444 PMC6199799

[12] Abengozar R , Sharma A , Sharma R . Gastric cancer: lessons learned from high‐incidence geographic regions. Journal of Gastrointestinal Oncology. 2021;12(Suppl 2):S350. 10.21037/jgo-2019-gi-05 34422399 PMC8343089

[13] Capelle LG , Van Grieken NC , Lingsma HF , Steyerberg EW , Klokman WJ , Bruno MJ , et al. Risk and epidemiological time trends of gastric cancer in Lynch syndrome carriers in The Netherlands. Gastroenterology. 2010;138(2):487–492. 10.1053/j.gastro.2009.10.051 19900449

[14] Isobe T , Hashimoto K , Kizaki J , Murakami N , Aoyagi K , Koufuji K , et al. Characteristics and prognosis of synchronous multiple early gastric cancer. World J Gastroenterol. 2013;19(41):7154–7159. 10.3748/wjg.v19.i41.7154 24222960 PMC3819552

[15] Abe S , Oda I , Minagawa T , Sekiguchi M , Nonaka S , Suzuki H , et al. Metachronous gastric cancer following curative endoscopic resection of early gastric cancer. Clin Endosc. 2018;51(3):253–259. 10.5946/ce.2017.104 28920420 PMC5997077

[16] Umar A , Boland CR , Terdiman JP , Syngal S , de la Chapelle A , Rüschoff J , et al. Revised Bethesda guidelines for hereditary nonpolyposis colorectal cancer (Lynch syndrome) and microsatellite instability. J Natl Cancer Inst. 2004;96(4):261–268. 10.1093/jnci/djh034 14970275 PMC2933058

[17] Vasen HF . Clinical diagnosis and management of hereditary colorectal cancer syndromes. J Clin Oncol. 2000;18(21 Suppl):81s–92s. 11060333

[18] Schouten JP , McElgunn CJ , Waaijer R , Zwijnenburg D , Diepvens F , Pals G . Relative quantification of 40 nucleic acid sequences by multiplex ligation‐dependent probe amplification. Nucleic Acids Res. 2002;30(12):e57. 10.1093/nar/gnf056 12060695 PMC117299

[19] Tomita N , Ishida H , Tanakaya K , Yamaguchi T , Kumamoto K , Tanaka T , et al. Japanese Society for Cancer of the colon and Rectum (JSCCR) guidelines 2020 for the clinical practice of hereditary colorectal cancer. Int J Clin Oncol. 2021;26(8):1353–1419. 10.1007/s10147-021-01881-4 34185173 PMC8286959

[20] Vasen HF , Blanco I , Aktan‐Collan K , Gopie JP , Alonso A , Aretz S , et al. Revised guidelines for the clinical management of Lynch syndrome (HNPCC): recommendations by a group of European experts. Gut. 2013;62(6):812–823. 10.1136/gutjnl-2012-304356 23408351 PMC3647358

[21] Tominaga S . Epidemiologic trends of stomach cancer in Japan and world. Nihon Rinsho. 2001;59(Suppl 4):5–12. 11424433

[22] Kinami S , Aizawa M , Yamashita H , Kumagai K , Kamiya S , Toda M , et al. The incidences of metachronous multiple gastric cancer after various types of gastrectomy: analysis of data from a nationwide Japanese survey. Gastric Cancer. 2021;24(1):22–30. 10.1007/s10120-020-01104-1 32780194 PMC7790780

[23] Kim J , Braun D , Ukaegbu C , Dhingra TG , Kastrinos F , Parmigiani G , et al. Clinical factors associated with gastric cancer in individuals with Lynch syndrome. Clin Gastroenterol Hepatol. 2020;18(4):830–837. 10.1016/j.cgh.2019.07.012 31319185 PMC6960373

[24] Saita C , Yamaguchi T , Horiguchi SI , Yamada R , Takao M , Iijima T , et al. Tumor development in Japanese patients with Lynch syndrome. PLoS One. 2018;13(4):e0195572. 10.1371/journal.pone.0195572 29672549 PMC5908237

[25] Yamaguchi T , Furukawa Y , Nakamura Y , Matsubara N , Ishikawa H , Arai M , et al. Comparison of clinical features between suspected familial colorectal cancer type X and Lynch syndrome in Japanese patients with colorectal cancer: a cross‐sectional study conducted by the Japanese Society for Cancer of the colon and Rectum. Jpn J Clin Oncol. 2015;45(2):153–159. 10.1093/jjco/hyu190 25404568

[26] Hashimoto T , Kurokawa Y , Takahashi T , Miyazaki Y , Tanaka K , Makino T , et al. Predictive value of MLH1 and PD‐L1 expression for prognosis and response to preoperative chemotherapy in gastric cancer. Gastric Cancer. 2019;22(4):785–792. 10.1007/s10120-018-00918-4 30617648

[27] Giardiello FM , Allen JI , Axilbund JE , Boland CR , Burke CA , Burt RW , et al. Guidelines on genetic evaluation and management of Lynch syndrome: a consensus statement by the US multi‐society task force on colorectal cancer. Gastroenterology. 2014;147(2):502–526. 10.1053/j.gastro.2014.04.001 25043945

[28] Zhao S , Chen L , Zang Y , Liu W , Liu S , Teng F , et al. Endometrial cancer in Lynch syndrome. Int J Cancer. 2022;150(1):7–17. 10.1002/ijc.33763 34398969

[29] Tanakaya K , Yamaguchi T , Ishikawa H , Hinoi T , Furukawa Y , Hirata K , et al. Causes of cancer death among first‐degree relatives in Japanese families with Lynch syndrome. Anticancer Res. 2016;36(4):1985–1989. 27069191

[30] Møller P , Seppälä TT , Bernstein I , Holinski‐Feder E , Sala P , Gareth Evans D , et al. Cancer risk and survival in path_MMR carriers by gene and gender up to 75 y of age: a report from the prospective Lynch syndrome database. Gut. 2018;67(7):1306–1316. 10.1136/gutjnl-2017-314057 28754778 PMC6031262

[31] Parry S , Win AK , Parry B , Macrae FA , Gurrin LC , Church JM , et al. Metachronous colorectal cancer risk for mismatch repair gene mutation carriers: the advantage of more extensive colon surgery. Gut. 2011;60(7):950–957. 10.1136/gut.2010.228056 21193451 PMC3848416

[32] Peng J , Wang Y . Epidemiology, pathology and clinical management of multiple gastric cancers: a mini‐review. Surg Oncol. 2010;19(4):e110–e114. 10.1016/j.suronc.2010.05.002 20566282

[33] Hamashima C . Update version of the Japanese guidelines for gastric cancer screening. Jpn J Clin Oncol. 2018;48(7):673–683. 10.1093/jjco/hyy077 29889263

[34] Vangala DB , Cauchin E , Balmaña J , Wyrwicz L , van Cutsem E , Güller U , et al. Screening and surveillance in hereditary gastrointestinal cancers: recommendations from the European Society of Digestive Oncology (ESDO) expert discussion at the 20th European Society for Medical Oncology (ESMO)/world congress on gastrointestinal cancer, Barcelona. Eur J Cancer. 2018;104:91–103. 10.1016/j.ejca.2018.09.004 30342310

[35] Kosuga T , Hiki N , Nunobe S , Noma H , Honda M , Tanimura S , et al. Feasibility and nutritional impact of laparoscopy‐assisted subtotal gastrectomy for early gastric cancer in the upper stomach. Ann Surg Oncol. 2014;21(6):2028–2035. 10.1245/s10434-014-3520-1 24558062

