# Supplementary material for: High risk of multiple gastric cancers in Japanese individuals with Lynch syndrome
Source: Ann Gastroenterol Surg. 2024 Apr 22;8(6):1008–16. doi: 10.1002/ags3.12809 (PMC11533028; doi:10.1002/ags3.12809)
Supplement: Supplementary file 1 — Table S1. [file AGS3-8-1008-s001.docx]

Supplementary Table 1. Genetic and clinical background of 31 probands with Lynch syndrome.

| Family number | Am II | rBG | | MMR gene | germline variant | InSiGHT Classification |
| --- | --- | --- | --- | --- | --- | --- |
| 1 | + | + | | *MLH1* | c.381_453del | class5 |
| 2 | + | + | | *MLH1* | c.381_453del | class5 |
| 3 | + | + | | *MLH1* | c.381_453del | class5 |
| 4 | - | + | | *MSH2* | c.203G>T | class5 |
| 5 | - | + | | *MLH1* | c.381_453del | class5 |
| 6 | + | + | | *MLH1* | c.381_453del | class5 |
| 7 | + | + | | *MLH1* | c.70_72delAAG | class5 |
| 8 | - | + | | *MSH2* | c.402C>T | class5 |
| 9 | - | + | | *MLH1* | c.381_453del | class5 |
| 10 | - | + | | *MSH2* | c.402C>T | class5 |
| 11 | - | + | | *MLH1* | c.545+1G>C | class5 |
| 12 | - | + | | *MLH1* | c.381_453del | class5 |
| 13 | + | + | | *MLH1* | c.381_453del | class5 |
| 14 | - | + | | *MSH2* | c.942+4A>G | class5 |
| 15 | + | + | | *MLH1* | c.381_453del | class5 |
| 16 | - | + | | *MLH1* | c.381_453del | class5 |
| 17 | - | + | | *MLH1* | c.381_453del | class5 |
| 18 | - | + | | *MLH1* | c.381_453del | class5 |
| 19 | - | + | | *MSH2* | c.1204C>T | class5 |
| 20 | - | + | | *MLH1* | c.1384A>T | class5 |
| 21 | - | + | | *MSH2* | c.1760-339_2458+483del | class5 |
| 22 | - | + | | *MLH1* | c.381_453del | class5 |
| 23 | - | + | | *MSH2* | c.942+3A>T | class5 |
| 24 | - | + | | *MSH2* | c.94delA | class5 |
| 25 | - | + | | *MSH2* | c.942+3A>T | class5 |
| 26 | - | + | | *MSH2* | c.328A>T | class5 |
| 27 | + | + | | *MSH2* | c.1861C>T | class5 |
| 28 | - | + | | *MSH6* | c.3962_3966dupGAGAA | class4 |
| 29 | + | | + | *MLH1* | c.1820_1823delinsAA | class5 |
| 30 | - | | + | *MLH1* | c.2200_2201dup | class4 |
| 31 | - | | + | *MSH2* | c.1861C>T | class5 |

Am II, Amsterdam II criteria. rBG, Revised Bethesda Guidelines. MMR, Mismatch Repair.
